# Editorial: Advances in artificial intelligence for early cancer detection and precision oncology

**DOI:** 10.3389/frai.2026.1911226

**Published:** 2026-07-28

**Authors:** Andreas Kanavos

**Affiliations:** 1 Department of Informatics, Ionian University, Corfu, Greece; 2 Ionio Panepistemio, Corfu, Greece

**Keywords:** artificial intelligence, clinical decision support, deep learning, early cancer detection, explainable artificial intelligence, medical imaging, personalized medicine, precision oncology

Artificial intelligence has become one of the most active areas of research in oncology. From image interpretation and pathology to molecular profiling and treatment planning, AI-based approaches are increasingly being explored as tools to support clinicians in making more informed decisions. When this Research Topic was launched, our aim was deliberately broad. We sought contributions that would reflect the many ways in which AI could contribute to earlier cancer detection and more personalized care, while also addressing some of the challenges that accompany its clinical adoption.

The response highlighted both the enthusiasm surrounding this field and its rapid evolution. In total, 22 manuscripts were submitted to this Research Topic, of which nine were accepted following peer review. Although these articles covered different cancer types, data modalities, and methodological approaches, they ultimately converged around a number of shared themes. Perhaps more importantly, they offered a realistic picture of where the field currently stands and where it may be heading.

One of the most evident themes was the continued effort to improve the early identification and characterization of cancer. Earlier detection remains one of the strongest arguments for integrating AI into clinical workflows. In this Research Topic, AI-assisted analysis of computed tomography scans identified malignant spinal lesions months before they were reported in routine practice, suggesting that subtle disease-related changes may be detectable before they become apparent to human observers. Similar ambitions were reflected in studies focusing on colorectal and skin lesions. Machine learning approaches improved the recognition of sessile serrated lesions during endoscopy, while a melanoma screening framework was specifically designed to minimize the risk of overlooking malignancies. Although these studies addressed different clinical problems, they shared a common objective: reducing missed diagnoses while preserving the opportunity for timely intervention.

At the same time, the contributions repeatedly reminded us that high performance alone is unlikely to guarantee clinical acceptance. Questions of trust, transparency, and interpretability have become increasingly difficult to ignore. Several studies addressed these issues directly. One combined radiological imaging with structured clinical information in an explainable multi-modal framework, demonstrating that predictive performance and interpretability do not necessarily represent competing priorities. Another explored the use of tissue symmetry patterns in colon histopathology, linking model explanations to morphological changes that pathologists already recognize in daily practice. Even studies based on more traditional machine learning techniques attempted to move beyond black-box predictions by identifying clinically meaningful features and presenting them in a way that could support decision-making. Taken together, these contributions suggest that explainability is no longer viewed as an optional addition but rather as an important component of clinically useful AI systems.

The Research Topic also reflected the increasing sophistication of methodological approaches. Several studies moved beyond single-source analyses by integrating information from multiple domains. Radiomics features derived from ultrasound images were combined with deep learning outputs and clinical variables to improve the differentiation of breast fibroepithelial tumors and to reduce unnecessary biopsies. Elsewhere, advances in foundation models were adapted to the specific demands of medical imaging, illustrating how developments originating outside healthcare can be reshaped for oncology applications. These studies differed substantially in their technical implementation, yet they shared a common goal: making better use of routinely available data to support more accurate and nuanced clinical assessments.

Another encouraging observation was the growing emphasis on clinical relevance. Across the articles, there was a clear effort to move beyond reporting isolated performance metrics. Multicenter validation strategies were increasingly adopted, and several authors attempted to evaluate how their methods might influence patient management. This was evident in studies demonstrating reductions in unnecessary invasive procedures and in work designed around clinically acceptable safety thresholds rather than optimizing accuracy alone. The final contribution extended this perspective even further by investigating whether deep learning models could help predict both EGFR mutation status and response to targeted EGFR inhibitor therapy in patients with metastatic non-small cell lung cancer. Such approaches point toward a future in which AI may contribute not only to diagnosis, but also to treatment selection and therapeutic planning.

Notably, one of the accepted articles stepped back from proposing a new model and instead examined the broader landscape of AI applications in cervical cytology. The review highlighted issues that many researchers and clinicians will recognize: limited external validation, increasing reliance on private datasets, inconsistent evaluation practices, and the persistent gap between promising research findings and routine clinical implementation. In many ways, these observations resonate with the broader messages emerging from the original studies included in this Research Topic.

Looking across all nine contributions, perhaps the most striking observation is that progress in oncology AI no longer seems to be defined solely by improvements in predictive performance. The field appears to be entering a more mature phase, one in which questions of reliability, transparency, validation, safety, and clinical impact receive equal attention. The challenge ahead is therefore not simply to build more sophisticated algorithms, but to develop systems that clinicians can understand, trust, and incorporate into everyday practice.

We hope that the studies presented in this Research Topic will encourage further collaboration between clinicians, computer scientists, engineers, and researchers working across disciplines. Advances in artificial intelligence hold considerable promise for cancer care, but their true value will ultimately be determined by whether they improve the experiences and outcomes of the patients they are intended to serve.

